# Beauty and Paintings: Aesthetic Experience in Patients with Behavioral Variant Frontotemporal Dementia When Viewing Abstract and Concrete Paintings

**DOI:** 10.3390/brainsci14050500

**Published:** 2024-05-15

**Authors:** Claire Boutoleau-Bretonnière, Catherine Thomas-Anterion, Anne-Laure Deruet, Estelle Lamy, Mohamad El Haj

**Affiliations:** 1 INSERM, CMRR Neurologie, CHU Nantes, Nantes Université, CIC 04, 44000 Nantes, France; 2 Laboratoire de Psychologie des Pays de la Loire, Nantes Université, Université Angers, LPPL, UR 4638, 44000 Nantes, France; 3 Laboratoire d’Etudes des Mécanismes Cognitifs, EA 3082, Université Lyon 2, 69500 Bron, France; catherine.thomas@univ-lyon2.fr; 4 Plein-Ciel, 69007 Lyon, France; 5 Clinical Gerontology Department, CHU Nantes, Bd Jacques Monod, 44093 Nantes, France; 6 Institut Universitaire de France, 75005 Paris, France; 7 LPPL—Laboratoire de Psychologie des Pays de la Loire, Faculté de Psychologie, Université de Nantes, Chemin de la Censive du Tertre, BP 81227, CEDEX 3, 44312 Nantes, France

**Keywords:** aesthetic, arts, behavioral variant frontotemporal dementia, frontotemporal dementia, neuroaesthetics

## Abstract

We assessed the aesthetic experience of patients with behavioral variant frontotemporal dementia (bvFTD) to understand their ability to experience feelings of the sublime and to be moved when viewing paintings. We exposed patients with bvFTD and control participants to concrete and abstract paintings and asked them how moved they were by these paintings and whether the latter were beautiful or ugly. Patients with bvFTD declared being less moved than control participants by both abstract and concrete paintings. No significant differences were observed between abstract and concrete paintings in both patients with bvFTD and control participants. Patients with bvFTD provided fewer “beautiful” and more “ugly” responses than controls for both abstract and concrete paintings. No significant differences in terms of “beautiful” and “ugly” responses were observed between abstract and concrete paintings in both patients with bvFTD and control participants. These findings suggest disturbances in the basic affective experience of patients with bvFTD when they are exposed to paintings, as well as a bias in their ability to judge the aesthetic quality of paintings.

## 1. Introduction

How do patients with behavioral variant frontotemporal dementia (bvFTD) judge paintings? We investigated this issue because we constantly encounter visual artworks in everyday life (e.g., advertisements and/or visual content on smartphone applications). Whether it is on the Internet, on television, in museums, or even in shopping malls, visual art is everywhere. In modern societies, people dedicate substantial time and money to visiting museums and buying art, even in a digital format with the advent of NFTs (non-fungible tokens). In addition to being a defining component of daily culture, viewing and judging art is a core component of one’s aesthetic experience. This experience has been investigated in the scientific and philosophical literature with early accounts of philosophers such as Kant who proposed that beautiful art could be recognized as such because the senses would respond universally to the artwork with a sensation of pleasure [1]. However, although the Kantian theory has been used extensively in the neuroaesthetic literature [1], the aesthetic response to art has not been shown to be universal [2]. Research suggests that, while initial affective judgments primarily recruit limbic and orbitofrontal structures, subsequent activation in the Default Mode Network only occurs in people who are highly moved by the artwork [2,3,4].

### 1.1. Aesthetic Experience in bvFTD

In this paper, we assessed the aesthetic experience in patients with bvFTD as this experience influences many aspects of life, from the enhancement of subjective well-being to making purchasing decisions [5,6]. We specifically assessed the ability of patients with bvFTD to experience feelings of the sublime and to be moved when viewing paintings, as well as their ability to judge their aesthetic quality. Since the aesthetic experience is a quintessential part of the human affective experience, we assessed how looking at paintings engages the aesthetic experience of patients with bvFTD. We assessed aesthetic processing in patients with bvFTD to better understand their subjective experience and affective functioning in general. According to the criteria of Rascovsky and Hodges [7], bvFTD is mainly characterized by deterioration of “personality, social comportment and cognition”. bvFTD hinders affective processing, resulting in apathy and a loss of empathy [7,8]. Disturbances in affective processing are a component of the prototypical presentation of bvFTD, and these disturbances can be covered by both Symptom A (i.e., early behavioral disinhibition) and Symptom C (i.e., early loss of sympathy or empathy) of bvFTD [7]. These affective disturbances have been reported by a body of research demonstrating pervasive changes in emotion processing, empathy, theory of mind, moral reasoning, and understanding of social rules [9,10,11,12]. Socio-affective disturbances in bvFTD can be associated with the atrophy of the brain’s frontotemporal areas [7,13]. These disturbances are critically involved in emotional and socio-affective processing [7,13]. Affective disturbances in bvFTD are also associated with global neural system disturbances, especially in the autonomic system, thus leading to disturbances in associating internal and external stimuli. This, in turn, leads to inappropriate behavioral responses [12,14,15]. As affective processing disturbances are a key feature in the symptomatology of bvFTD and may have consequences for patients’ ability to engage or respond socially, we assessed their affective processing by asking them about their feelings when viewing paintings.

### 1.2. Previous Research on Aesthetic Experience in bvFTD

Aesthetic experience in patients with bvFTD was evaluated in a study by Boutoleau-Bretonnière and Bretonnière [16], who exposed patients with bvFTD to abstract paintings. Patients were invited to tell how moved they were by the paintings. Patients were also invited to make aesthetic judgments by deciding whether the paintings were beautiful or ugly. The results demonstrated that bvFTD patients were little moved by the paintings compared to control participants (*p* < 0.001). Regarding the aesthetic dimension, the paintings were considered rather ugly by the patients (*p* < 0.001). These results suggest a hampered emotional and aesthetic experience as well as a negative aesthetic bias in patients with bvFTD. While the study of Boutoleau-Bretonnière et al. was the first to assess the aesthetic experience of patients with bvFTD when viewing paintings, it only included abstract artworks [16]. This diminished aesthetic experience may therefore be attributed to the abstract nature of the stimuli. Concrete paintings may elicit an enhanced aesthetic experience in patients with bvFTD, as concrete scenes (e.g., natural scenes such as sunsets) may trigger more emotions than abstract stimuli. The contents of concrete scenes may also be less difficult to process and identify than abstract scenes, which may enhance the affective experience of patients with bvFTD. We, therefore, extended the study of Boutoleau-Bretonnière and Bretonnière [16] by assessing the aesthetic experience of patients with bvFTD when viewing both abstract and concrete paintings. 

In line with previous research, we assessed the aesthetic experience of patients with bvFTD by inviting them to describe their aesthetic experience (i.e., whether they were a little or very moved) when viewing paintings. We also invited them to judge these paintings, i.e., to decide whether they were beautiful or ugly. We took the beautiful/ugly judgment into account as beauty has been considered to be a key concept of aesthetics [17,18], especially for visual arts [19]. Psychological theories have also emphasized how beauty plays a pivotal role in aesthetic experience and art judgment [20,21]. The fluency theory of aesthetic pleasure [22] posits that processing beauty is at the center of the aesthetic experience. Philosophers such as Hume [23] have also suggested that beauty is the essence of the aesthetic experience. In light of this literature, we invited patients with bvFTD to decide whether abstract and concrete paintings were beautiful or not.

### 1.3. Objectives and Hypotheses

Affective disturbances can be considered a key feature of bvFTD [7,9,10,11,12]. However, we assessed whether looking at paintings would engage the aesthetic experience of patients with bvFTD as well as their reflexive judgment mechanisms despite their affective disturbances. We, therefore, exposed patients with bvFTD and control participants to both concrete and abstract paintings using a computerized task. We asked them how moved they were by the paintings and to judge whether the latter were beautiful or ugly. Considering the affective disorders in bvFTD [7,9,10,11,12], we hypothesized that patients with bvFTD would be less moved by the paintings and would judge them as less beautiful than the controls. We also hypothesized that patients with bvFTD would be more moved by concrete paintings and judge them as more beautiful compared with abstract paintings.

## 2. Method

### 2.1. Participants

**Figure 1:**
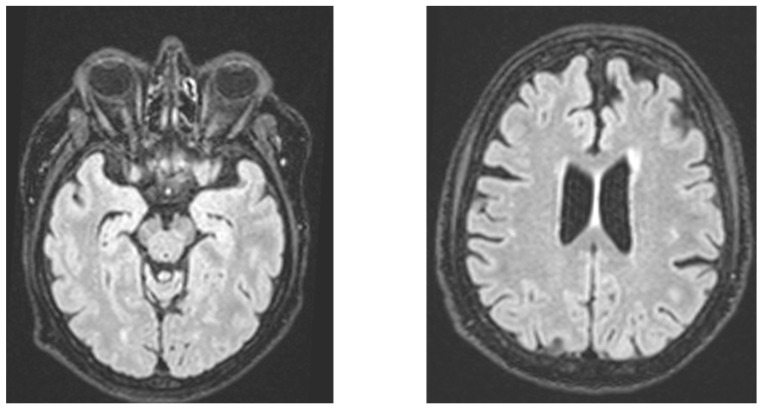
Illustration of the atrophy in a participant with bvFTD.

Sixteen patients (ten men and six women, *M* age = 68.4 years, *SD* = 7.90, *M* years of formal education = 10.4, *SD* = 4.90) were recruited from the Memory Clinic Center at the Neurology Department of the Hospital of Nantes, France, and the diagnosis of possible bvFTD was made based on Rascowsky’s criteria, including neuroimaging-confirmed atrophy (an illustration is provided in Figure 1). We also recruited 16 control participants (seven men and nine women, *M* age = 67.91 years, *SD* = 7.90, *M* years of formal education = 11.3, *SD* = 3.21) who were mostly composed of caregivers of patients and were free of any neurological, affective, or psychiatric disorder. Control participants were matched with patients with bvFTD according to age [*t*(30) = 1.64, *p* = 0.11], sex [*X*^2^ (1, *N* = 59) = 1.13, *p* = 0.29] and educational level [*t*(30) = 1.16, *p* = 0.25]. The control participants (*M* = 29.56, *SD* = 0.41) obtained higher scores on the Mini Mental State Exam than the patients (*M* = 21.13, *SD* = 3.02) [*t*(30) = 11.13, *p* < 0.001]. This study’s protocol was performed in compliance with the Code of Ethics of the World Medical Association (Declaration of Helsinki). All patients and caregivers gave written informed consent prior to their participation in this study.

### 2.2. Procedure

#### 2.2.1. Materials

**Figure 2:**
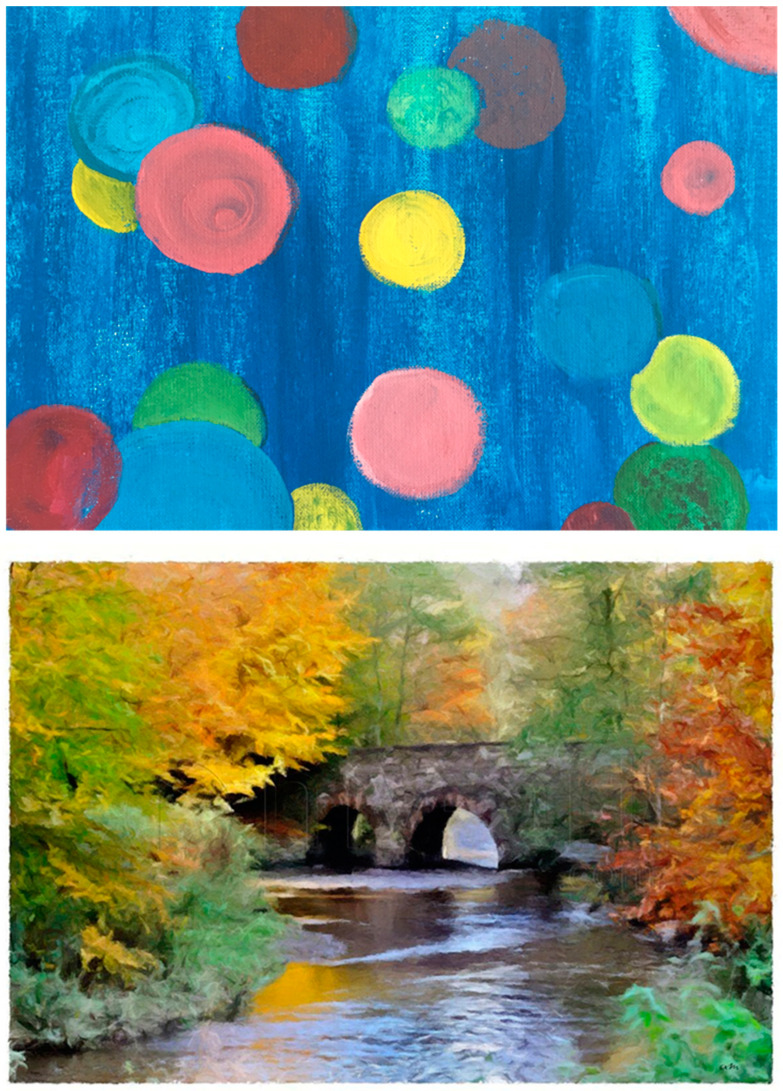
An illustration of an abstract (**above**) and a concrete (**below**) painting.

The task was developed in collaboration with the Emotion Task Force group (Laboratory EA 3082, University Lyon 2) for abstract paintings and with the “Laboratoire de Psychologie des Pays de la Loire” (LPPL—EA 4638) for concrete paintings. Materials involved 16 concrete and 16 abstract paintings (see Figure 2 for an illustration). All were non-famous and found online on museum websites. By using paintings from museums or private collections, we ensured that the paintings responded to a consensual agreement about their value. However, prior to choosing the pieces and in order to ensure that the study would not be influenced by familiarity, we invited 50 independent control participants with no enhanced knowledge of art to judge the familiarity of the paintings. We only retained paintings considered non-familiar. 

#### 2.2.2. Aesthetic Experience Task

The task was conducted using the Psyscope XB57D application (Macintosh) on a Mac Book Pro laptop computer with 15.4-inch screen. Participants were informed in advance that the aim of the task was to assess what they felt when viewing the paintings. They were also informed that they were free to give any answer they wanted without seeking to identify logical links. When the participants were ready, they were exposed to each of the 16 abstract and 16 concrete paintings. The order of presentation was random for each participant. For each painting, the participants had to declare, with no time limit, how much they were moved by it. Their response was given by touching one of nine keys on the keyboard, with each key representing a number ranging from zero (“not at all”) to nine (“extremely”). The participants then had to decide, with no time limit, whether the painting was “beautiful” or “ugly” using a green or red key, respectively.

#### 2.2.3. Statistical Analysis

We compared the mean of “feeling moved” responses with the maximum score of nine points between patients with bvFTD and control participants using independent *t*-tests. For each population, we compared abstract and concrete tables using paired *t*-tests. We carried out the same analysis for the mean of “beautiful” responses with a maximum score of 16 points, and for the mean of “ugly” responses with a maximum score of 16 points. The higher the “beautiful” scores were, the lower the “ugly” scores were, and vice versa, as participants had to choose between these two categories. We applied *t*-tests after verifying normal distribution using Shapiro–Wilk tests. We calculated effect sizes by using Cohen’s *d* (0.20 = small, 0.50 = medium, 0.80 = large) [24]. For all tests, the significance level was set at *p* < 0.05.

### 2.3. Results

#### 2.3.1. “A Little Touched” Experience in Patients with bvFTD

**Figure 3:**
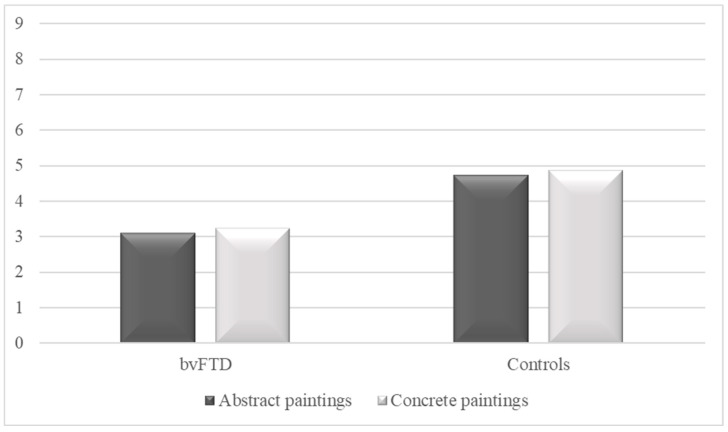
Means of “moved” responses to paintings (0 = not at all, 9 = extremely) in patients with behavioral variant of frontotemporal dementia (bvFTD) and control participants. Note: responses ranged from zero (not at all) to nine (extremely).

As illustrated in Figure 3, patients with bvFTD declared being less moved (*M* = 3.13, *SD* = 1.15) than control participants (*M* = 4.75, *SD* = 1.30) by abstract paintings (*t*(30) = 3.76, *p* = 0.001, Cohen’s *d* = 1.33). They also declared being less moved (*M* = 3.25, *SD* = 1.29) than the controls (*M* = 4.88, *SD* = 1.59) by concrete paintings (*t*(30) = 3.18, *p* = 0.003, Cohen’s *d* = 1.12). In patients with bvFTD, no significant differences were observed between the “moved” responses to concrete and abstract paintings (*t*(15) = 0.44, *p* = 0.67, Cohen’s *d* = 0.10). The same observation was made for the control participants (*t*(15) = 0.38, *p* = 0.71, Cohen’s *d* = 0.08).

#### 2.3.2. Few “Beautiful” Responses in Patients with bvFTD

**Figure 4:**
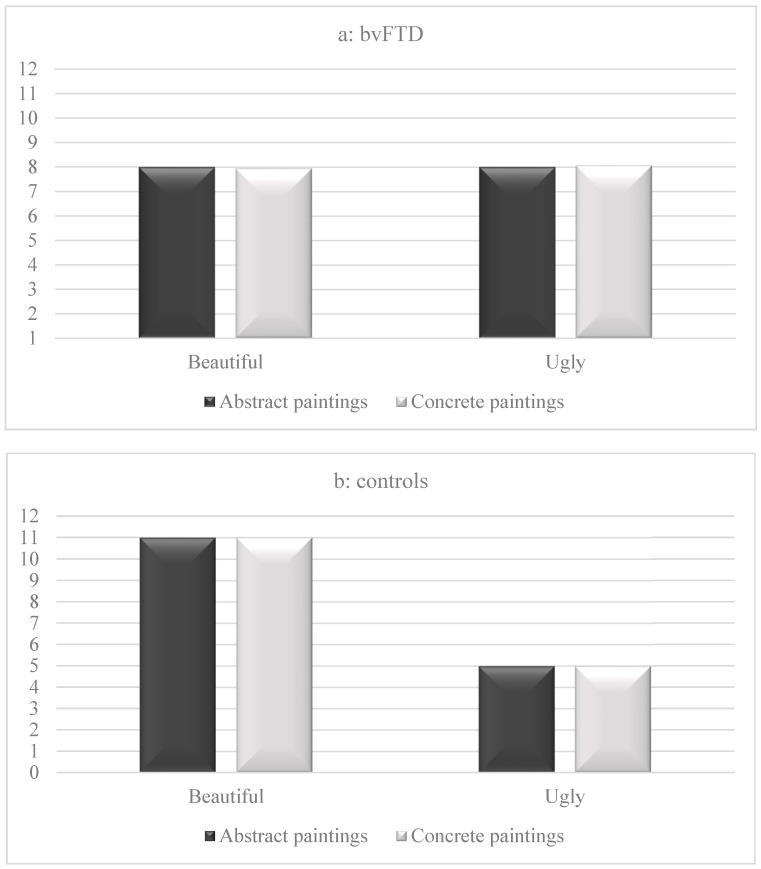
Means of “beautiful” and “ugly” responses to concrete and abstract paintings in patients with behavioral variant of frontotemporal dementia (bvFTD) and control participants. Note: the maximum number of “beautiful” or “ugly” responses was 16 points.

As illustrated in Figure 4, patients with bvFTD gave fewer “beautiful” responses (*M* = 8.00, *SD* = 3.93) than control participants (*M* = 11.25, *SD* = 2.24) to abstract paintings (*t*(30) = 2.87, *p* = 0.007, Cohen’s *d* = 1.0). They also provided fewer “beautiful” responses (*M* = 7.94, *SD* = 3.59) than the controls (*M* = 11.06, *SD* = 2.26) to concrete paintings (*t*(30) = 2.95, *p* = 0.006, Cohen’s *d* = 1.04). In patients with bvFTD, no significant differences were observed between the “beautiful” responses to concrete and abstract paintings (*t*(15) = 0.12, *p* = 0.91, Cohen’s *d* = 0.03), as was observed in control participants (*t*(15) = 0.30, *p* = 0.77, Cohen’s *d* = 0.07).

Patients with bvFTD gave more “ugly” responses (*M* = 8.00, *SD* = 3.93) than control participants (*M* = 4.88, *SD* = 2.31) to abstract paintings (*t*(30) = 2.74, *p* = 0.01, Cohen’s *d* = 1.01). They also gave more “ugly” responses (*M* = 8.06, *SD* = 3.58) than the controls (*M* = 4.94, *SD* = 2.30) to concrete paintings (*t*(30) = 2.95, *p* = 0.006, Cohen’s *d* = 1.04). No significant differences were observed in patients with bvFTD between the “ugly” responses to concrete and abstract paintings (*t*(15) = 0.12, *p* = 0.91, Cohen’s *d* = 0.03). The same observation was made in control participants (*t*(15) = 0.10, *p* = 0.92, Cohen’s *d* = 0.03). Note that no significant correlations were observed between scores on the Mini Mental State Exam and any of the aesthetic scores (*p* > 0.1). 

## 3. Discussion

We assessed the aesthetic experience of patients with bvFTD when viewing abstract and concrete paintings. They declared being less moved than control participants by both abstract and concrete paintings. No significant differences regarding the experience of being moved were observed between abstract and concrete paintings in both patients with bvFTD and controls. In terms of beauty judgment, patients with bvFTD gave fewer “beautiful” and more “ugly” responses than control participants for both abstract and concrete paintings. No significant differences in “beautiful” and “ugly” responses were observed between abstract and concrete paintings in both patients with bvFTD and controls.

Patients with bvFTD declared being less moved than control participants by both abstract and concrete paintings. They gave fewer “beautiful” and more “ugly” responses to the paintings than controls. The absence of difference between abstract and concrete paintings is contrary to what was expected in a study [16] with abstract paintings only, according to which bvFTD patients may experience cognitive issues as spectators owing to their difficulties in dealing with abstraction. Our findings suggest a globally compromised aesthetic experience in patients with bvFTD. This is supported by research showing affective disturbances in bvFTD [7,9,10,11,12]. The “a little moved” responses in patients with bvFTD may be attributed to underlying disturbances in affective experience (e.g., arousal reaction), which may in turn lead to a bias in judging the aesthetic quality of paintings. This bias may also be attributed to a hampered ability to reflect on the “beautiful” and the “ugly”, as this requires enhanced cognitive resources. Aesthetic judgment requires reflection and deliberation over the basic emotional reaction when viewing art [25,26]. These advanced cognitive processes may be compromised in patients with bvFTD owing to their general cognitive decline.

By assessing both the “moved” and “beauty” experience of patients with bvFTD in reaction to abstract and concrete paintings, our study contributes to the literature on socioemotional disturbances in bvFTD. It distinguishes between the basic affective reaction (i.e., being moved) of patients with bvFTD and their ability to reflect on the beauty of paintings (i.e., ugly vs. beautiful responses). Research on socioemotional disturbances in bvFTD should differentiate between these two levels: the basic affective reaction may reveal the nature of patients’ physiological and behavioral responses when encountering affective stimuli, while the reflective level may reveal patients’ ability to identify and interpret affective stimuli. In our study, we examined both the basic affective reaction and the reflective judgment regarding concrete and abstract paintings, and we observed similar reactions to both types of stimuli. Contrary to our hypothesis, patients with bvFTD showed similar reactions towards concrete and abstract paintings, as was observed in control participants. These findings are interesting as they show that abstract and concrete paintings may trigger a similar affective experience in both normal and pathological populations.

At the neural level, our study contributes to the field of neuroaesthetics, which examines the neural and behavioral basis of aesthetic experiences [27,28]. Neuroaesthetic research has identified the brain circuits that support aesthetic experiences, including subcortical reward circuitry, sensory and motor pathways, default-mode networks, and, critically, prefrontal cortex pathways [2,3]. It has also demonstrated the involvement of the dorsolateral, ventrolateral, and anterior medial prefrontal cortexes in processing and judging art [29]. The dorsolateral prefrontal cortex has been shown to be involved in processing the beauty of geometric shapes, and the ventromedial prefrontal cortex in processing several aspects of beauty [30,31]. The aesthetic reactions of patients with bvFTD may be attributed to the atrophy of the prefrontal cortex induced by the condition [32,33].

Although our findings suggest a hampered aesthetic experience or even a negative aesthetic bias in patients with bvFTD, an alternative yet controversial explanation may be that these patients have difficulties expressing their aesthetic experience, thus resulting in the “a little moved” and “ugly” responses in our study. Also, the patients may tend to declare being “a little moved” by paintings as a reflection of their self-images (e.g., having a medical condition, being physically impacted by this condition, and/or not being very concerned by the environment). While this assumption needs to be tested empirically, it paves the way for interesting therapeutic avenues, such as assessing whether art therapy may enhance the aesthetic experience of patients with bvFTD in general and their self-images.

One potential limitation of our paper may be the absence of an assessment of apathy and theory of mind. While the bvFTD patients in our study all exhibited apathy, as per the diagnosis criteria, this observation was made during clinical interviews rather than through a specific assessment. It would have been valuable to incorporate an apathy assessment to explore potential relationships with disturbances in the basic affective experience of patients with bvFTD when exposed to paintings. Similarly, the same holds true for the theory of mind; disturbances in the basic affective experience of patients with bvFTD when exposed to paintings may be associated with disruptions in the affective theory of mind. However, it should be noted that, in our study, we had to minimize the assessment load as much as possible to mitigate the risk of fatigue, disinhibition, and lack of motivation, as may be observed in some patients with bvFTD. In a similar vein, this study included only 16 patients because bvFTD is a rare condition [34,35], with the sample size defined as the maximum number of patients willing to participate during the study period, spanning nearly two years. Our preliminary study should nonetheless be replicated in a larger sample of patients.

## 4. Conclusions

The last two decades have seen a development of neuroaesthetic research and a renewed focus on art viewing. This interest is concomitant with the emergence of new integrative methodologies between neurological and cognitive assessments. Our study contributes to this literature by shedding light on the assessment of aesthetic experience in bvFTD and might lead to new art therapy avenues in the field of bvFTD research.

## Data Availability

Raw data are provided in Appendix A.

## Appendix A

**Table A1:** Raw data.

	Touched/Abstract	Touched/Concrete	Beautiful/Abstract	Beautiful/Concrete	Ugly/Abstract	Ugly/Concrete
bvFTD	3	2	16	14	0	2
bvFTD	2	4	9	11	7	5
bvFTD	3	3	14	12	2	4
bvFTD	2	2	13	9	3	7
bvFTD	3	3	8	9	8	7
bvFTD	5	7	8	9	8	7
bvFTD	2	3	3	5	13	11
bvFTD	3	2	3	7	13	9
bvFTD	4	5	11	14	5	2
bvFTD	5	4	6	5	10	11
bvFTD	3	3	8	9	8	7
bvFTD	2	3	5	4	11	12
bvFTD	4	3	8	6	8	10
bvFTD	5	3	8	7	8	9
bvFTD	2	2	5	4	11	12
bvFTD	2	3	3	2	13	14
Controls	5	6	11	9	7	7
Controls	6	5	9	11	7	5
Controls	4	4	8	10	8	6
Controls	5	6	11	9	5	7
Controls	3	4	9	11	7	5
Controls	4	5	10	14	6	2
Controls	5	4	16	14	0	2
Controls	6	8	12	15	4	1
Controls	3	3	12	12	4	4
Controls	4	3	9	7	7	9
Controls	7	6	10	10	6	6
Controls	5	7	11	8	5	8
Controls	3	4	14	12	2	4
Controls	4	2	12	13	4	3
Controls	5	6	15	10	1	6
Controls	7	5	11	12	5	4
